# Genetic Divergence of Two *Sitobion avenae* Biotypes on Barley and Wheat in China

**DOI:** 10.3390/insects11020117

**Published:** 2020-02-11

**Authors:** Da Wang, Xiaoqin Shi, Deguang Liu, Yujing Yang, Zheming Shang

**Affiliations:** 1 State Key Laboratory of Crop Stress Biology for Arid Areas, Northwest A&F University, Yangling 712100, Shaanxi, China; wangda@nwsuaf.edu.cn (D.W.); yyj214812579@126.com (Y.Y.); shangzheming2011@sina.com (Z.S.); 2 College of Plant Protection, Northwest A&F University, Yangling 712100, Shaanxi, China; 3 Department of Foreign Languages, Northwest A&F University, Yangling 712100, Shaanxi, China; sxq-shi@nwsuaf.edu.cn

**Keywords:** genetic differentiation, host-associated differentiation, genetic structure, genotype, biotype development, geographic populations

## Abstract

Host plant affinity and geographic distance can play critical roles in the genetic divergence of insect herbivores and evolution of insect biotypes, but their relative importance in the divergence of insect populations is still poorly understood. We used microsatellite markers to test the effects of host plant species and geographic distance on divergence of two biotypes of the English grain aphid, *Sitobion avenae* (Fabricius). We found that clones of *S. avenae* from western provinces (i.e., Xinjiang, Gansu, Qinghai and Shaanxi) had significantly higher genetic diversity than those from eastern provinces (i.e., Anhui, Henan, Hubei, Zhejiang and Jiangsu), suggesting their differentiation between both areas. Based on genetic diversity and distance estimates, biotype 1 clones of eastern provinces showed high genetic divergence from those of western provinces in many cases. Western clones of *S. avenae* also showed higher genetic divergence among themselves than eastern clones. The Mantel test identified a significant isolation-by-distance (IBD) effect among different geographic populations of *S. avenae*, providing additional evidence for a critical role of geography in the genetic structure of both *S. avenae* biotypes. Genetic differentiation (i.e., *F*_ST_) between the two biotypes was low in all provinces except Shaanxi. Surprisingly, in our analyses of molecular variance, non-significant genetic differentiation between both biotypes or between barley and wheat clones of *S. avenae* was identified, showing little contribution of host-plant associated differentiation to the divergence of both biotypes in this aphid. Thus, it is highly likely that the divergence of the two *S. avenae* biotypes involved more geographic isolation and selection of some form than host plant affinity. Our study can provide insights into understanding of genetic structure of insect populations and the divergence of insect biotypes.

## 1. Introduction

Many insect species are serious pests on various horticultural plants and agricultural crops. In order to develop ecologically based management programs, it is of great importance to understand the genetic diversity and population structure of insect pests. Genetic diversity and divergence among populations are essential in enabling insect species to respond rapidly to evolutionary challenges, thus having significant impacts on the adaptability of insects under constantly changing environmental conditions [1,2,3]. During the long term evolutionary process, insect species could have experienced genetic differentiation and diversification from combined effects of mutation, selection, gene flow, and genetic drift [4,5,6,7]. In this process, gene flow among populations could have been prevented by some environmental barriers, and local selection could be in turn accelerated, thus promoting changes in genetic structure of populations [6,7,8]. Demographic histories involving events of inbreeding and migration can also affect genetic structure of pest insect populations [9,10]. Many studies have shown that different geographic populations can show patterns of IBE (isolation by environment) or IBD (isolation by distance) (IBD) [11,12,13]. In addition, host plants are another significant factor influencing genetic structure of insect populations, since host-plant associated differentiation appears to be a common phenomenon for insect populations [7,14,15,16]. Indeed, various biotypes (usu. host plant associated populations) have been constantly discovered in many pest insects like the pea aphid (*Acyrthosiphon pisum*), Hessian fly (*Mayetiola destructor*), black currant leaf midge (*Dasineura tetensi*), and soybean aphid (*Aphis glycines*) [17,18,19,20].

Different insect biotypes can be distinguished by their characteristic response patterns (i.e., differential life-history traits or fitness) on different plants or different varieties of the same plant [21,22]. In particular, aphid species are prone to evolve different biotypes on variable plants because of their unique characteristics like common occurrence of local adaptation and phenotypic plasticity [23,24,25], induction of significant plant responses with saliva [26,27,28], and harboring various primary and secondary endosymbionts (e.g., *Buchnera*, *Regiella* and *Hamiltonella*) [29,30,31]. About half of all the insect species with known biotypes are aphids [32]. Indeed, the evolution of biotypes has been found to occur in at least 17 species of aphids, such as the pea aphid (*A. pisum*), greenbug (*Schizaphis graminum*), and Russian wheat aphid (*Diuraphis noxia*) [17,33,34,35]. Since the late 1990s, there have been a great number of studies that use DNA markers to examine geographic or host-plant associated differentiation of various aphids [2,36,37,38,39]. But the relative importance of geography and host plant use on the biotype divergence of aphid populations, as well as genetic relationships among different aphid biotypes, is still not well understood [22,36,37,38]. 

Here, we use the English grain aphid, *Sitobion avenae* (Fabricius) (Hemiptera: Aphididae), as a model to address this issue. This aphid can use and survive on various cereal crops and numerous wild grasses [15,40,41]. Multiple biotypes of this aphid have been discovered based on their unique response patterns on different barley and wheat varieties [22]. Biotypes 1 and 2 of *S. avenae* were found to be the most common in China [22]. Microsatellite markers have been widely used in many studies on population genetics of aphids, involving genotypic diversity [2,42], host range expansion [43], and host plant specialization [14,44], and identification of migration routes [2,45]. In this study, we use microsatellites to interpret the population genetics of the two predominant *S. avenae* biotypes, which can be critical for relevant crop breeding and integrated management programs. Specifically, our objectives are: (1) to examine genetic diversity and structure of the two *S. avenae* biotypes; (2) to explore genetic differentiation and relationship between both biotypes of *S. avenae*; and (3) to assess the importance of geography and host plant use in explaining differentiation of *S. avenae* biotypes.

## 2. Materials and Methods

### 2.1. Insect Sampling 

Apterous adults of *S. avenae* were collected on wheat (*Triticum aestivum* L.) and barley (*Hordeum vulgare* L.) from nine provinces of China (Table 1). We randomly selected *S. avenae* samples from more than five wheat or barley fields (cultivars and pest control measures unknown) at the same location. In order to minimize the probability of collecting identical clones, apterous aphid individuals were taken at a distance of >10 m [7,46]. A minimum of 50 (up to 137) aphid clones were collected in each province during April–July 2016. 

### 2.2. Aphid Genotyping

Six microsatellite loci (i.e., Sm10, Sm17, S17b, S5.L, S4Σ, and Sm12) were used to genotype all collected individuals of *S. avenae* (Supplementary Table S1) [7,47,48]. For each microsatellite locus, three primers (synthesized by Sangon Biotech Co., Ltd., Shanghai, China) were utilized, including a sequence-specific reverse primer, a sequence-specific forward primer with M13 (−21) tail added at its 5′ end, and a M13 (−21) primer fluorescent-labeled with FAM (i.e., 6-carboxy-fluorescine) [49]. The 25 μL-volume PCR reactions contained 12.5 μL 2 × Taq Master Mix (TaKaRa Biomedical Technology Co. Ltd., Beijing, China), 1.5 μL template DNA (15–25 ng/μL), 1 μL forward primer (10 μM), 2 μL reverse primer (10 μM), 1.5 μL M13 (−21) primer (10 μM), and 6.5 μL ddH_2_O. The PCR amplifications were conducted using a CFX-96 thermocycler (Bio-Rad Laboratories Inc., Hercules, CA, USA) with a temperature program as follows: 94 °C for 2 min, followed by 30 cycles (94 °C for 30 s, 30 s at the annealing temperature of each primer, 72 °C for 30 s), then 8 cycles (94 °C for 30 s, 53 °C for 45 s, and 72 °C for 45 s), then the last step at 72 °C for 10 min. The length of all PCR products was determined with the automated DNA sequencer ABI3730XL (Applied Biosystems, Foster City, CA, USA). Genotypes of *S. avenae* were distinguished with GENCLONE 2.0 [50]. Detailed information on these genotypes was included in Supplementary Materials (i.e., Table S2), including geography, host plant and allele sizes. 

### 2.3. Biotype Identification

The identification of biotypes 1 and 2 in these provinces was conducted as described in [22]. Briefly, dozens of selected barley and wheat varieties were screened for resistance against *S. avenae* by using life-history bioassays. All collected *S. avenae* genotypes were then tested for their response profiles on resistant barley/wheat varieties identified in the above-mentioned screening life-history tests and three susceptible controls (i.e., Aikang 58, Mingxian 169, and Xinong 979). Based on these tests, it was found that five wheat/barley (i.e., wheat: Zhong 4 wumang, and 186.TM12-34; barley: Dulihuang, Zaoshu No.3, and Xiyin No.2) varieties could be used to distinguish between *S. avenae* biotypes. Biotype 1 was non-virulent on the five wheat/barley varieties mentioned above, whereas biotype 2 was characterized by its virulence on the barley (*Hordeum vulgare* L.) cultivar Zaoshu No.3. 

### 2.4. Data Analysis

The software GenAlEx version 6.5 was utilized to analyze the following parameters: number of alleles (Na), number of effective alleles (Ne), observed heterozygosities (Ho), expected heterozygosities (He), and Shannon’s information index (I) [51]. We used the software FSTAT Version 2.9.3.2 [52] to determine and compare the estimates of gene diversity (H_S_) and allelic richness (AR) for biotypes 1 and 2. Using this software, Hardy–Weinberg equilibrium (HWE) was also tested for *S. avenae* populations. In all the above-mentioned analyses, we used data sets with only a single representative of each multi-locus genotype (MLG) per population following [2,47]. 

The software ARLEQUIN (version 3.5.1.2) was used to conduct the analysis of molecular variance (AMOVA) [53]. The software ARLEQUIN was also used to assess the pairwise fixation index (*F*_ST_) and its significance with 9999 bootstraps. Effects of isolation by distance (IBD) were evaluated (9999 permutations) with the Mantel test in the program GenAlEx (version 6.5) [51], for which matrices for both genetic *F_ST_*/(1-*F_ST_*) and geographic distances between different populations of *S. avenae* were used. Based on Nei’s standard genetic distance (Ds), we used MEGA 5 to establish a neighbor-joining (NJ) tree of *S. avenae* clones [54]. Ds between clones of *S. avenae* was calculated by using the microsatellite analyzer (MSA) version 3.15 [55]. In order to visualize genetic variation among *S. avenae* clones, a principal coordinate analysis (PCoA) was conducted with GenAlEx (version 6.5) [51]. The genetic structure of *S. avenae* biotypes was evaluated with the Bayesian clustering program in STRUCTURE version 2.3.3 [56]. In this analysis, the number of genetic clusters (K) was set from 1 to 13; 20 replicates were performed for each K; in each replicate, a burn-in period of 10,000 iterations and 100,000 Markov chain Monte Carlo (MCMC) iterations were conducted. The most probable value of genetic clusters (K) was identified with the Evanno method in STRUCTURE HARVESTER [57,58]. The genetic assignment software BayesAss (version 1.3) was used to examine the direction and magnitude (m, the proportion of migrant individuals in each population) of gene flow between *S. aveane* clones [45]. Based on the posterior probability distribution, the proportion of immigrants from one population to another can be determined with a fully Bayesian MCMC (Markov chain Monte Carlo) resampling method [2,45].

## 3. Results

### 3.1. Geographic Distribution and Genetic Diversity of Biotypes 1 and 2

**Figure 1:**
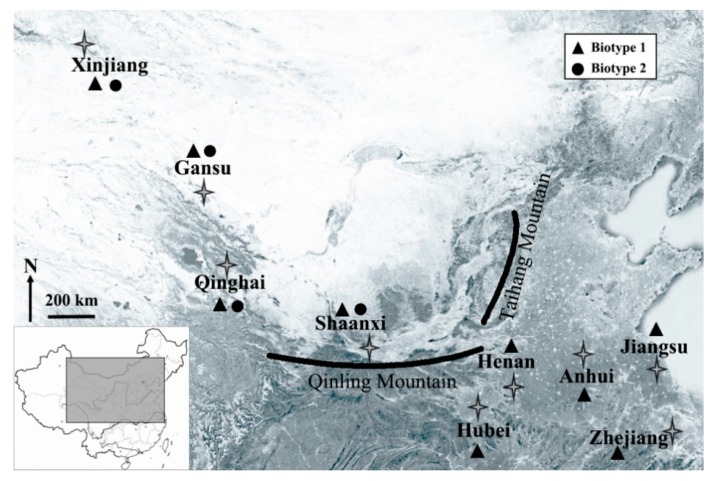
Collection of *Sitobion avenae* biotypes 1 and 2 from nine provinces of China.

Cones of biotype 1 were distributed over all provinces sampled in this study, whereas those of biotype 2 occurred only in western provinces (i.e., Shaanxi, Qinghai, Gansu, and Xinjiang) (Figure 1). The numbers of genotypes identified for biotype 1 in each province ranged from eight to 49 (Table 2), and those for biotype 2 ranged from five to six in each province, indicating higher genotypic diversity for biotype1 compared with biotype 2. 

Mean numbers of alleles (Na) ranged from 1.6 for biotype 2 collected from Gansu (GS2) to 11.33 for biotype 1 of Qinghai (QH1) (Table 2), whereas the number of effective alleles (Ne) varied from 0.9 for GS2 to 4.8 for biotype 1 of Shaanxi (SX1). Gene diversity (Hs) was lowest (i.e., 0.651) for biotype 1 of Anhui (AH1), and highest (i.e., 0.801) for SX1. Allelic richness (AR) varied from 3.74 for biotype 1 of Xinjiang (XJ1) to 5.26 for biotype 1 of Jiangsu (JS1). The Shannon’s information index (I) ranged from 1.01 for GS2 to 1.78 for QH1. The values of Ho and He fell in the range of 0.06 for GS2 to 0.77 for biotype 2 of Shaanxi (SX2), and 0.06 for GS2 to 0.75 for QH1, respectively. Based on all six microsatellite loci, significant deviation from HWE (HWE—*p* < 0.05) was found for all geographic populations but Anhui and Henan (Table S3).

**Figure 2:**
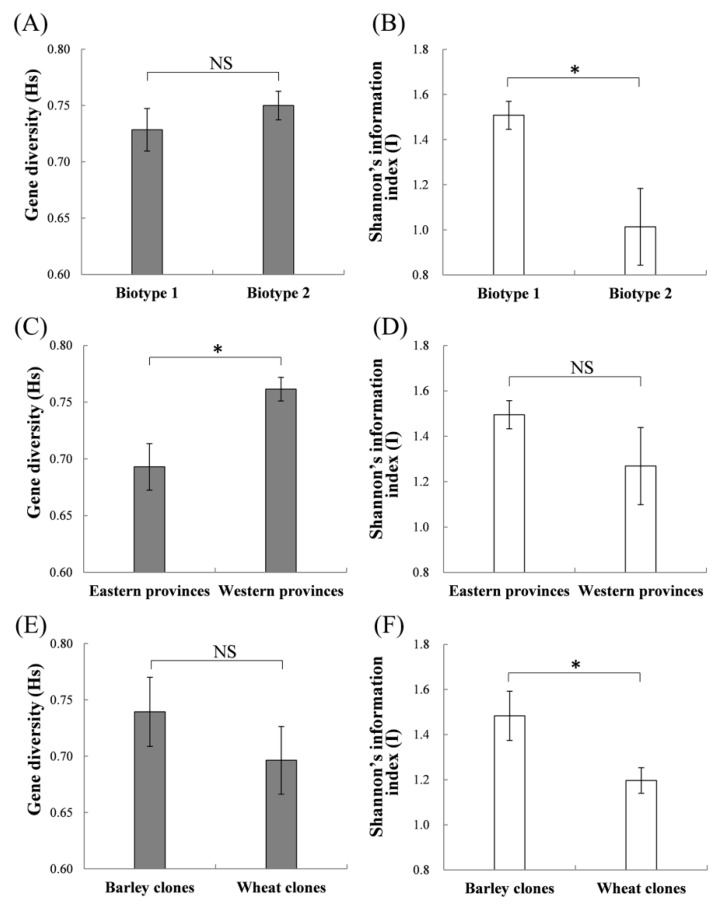
Comparisons of estimates for gene diversity (Hs) and Shannon’s information index (I). (**A**,**B**): between biotypes 1 and 2; (**C**,**D**): between eastern and western provinces; (**E**,**F**): between wheat and barley clones; eastern provinces include Anhui, Hubei, Henan, Jiangsu, and Zhejiang; western provinces include Gansu, Qinghai, Shaanxi, and Xinjiang; (*, significant differences at the *p* < 0.05 level; NS, non-significant).

Biotype 1 showed a significantly higher I (1.51 ± 0.06) than biotype 2 (1.01 ± 0.17) (Figure 2B; *t* = 2.542, *p* = 0.027), indicating higher genetic diversity for biotype 1 compared to biotype 2. A significantly higher I was also detected for barley clones (1.48 ± 0.11) compared with wheat clones (1.20 ± 0.06) (Figure 2F; *t* = 2.402, *p* = 0.036). Aphid clones of western provinces (0.76 ± 0.01) showed a higher gene diversity (Hs) than those of eastern provinces (0.69 ± 0.02) (Figure 2C; *t* = 2.977, *p* = 0.024). This result might be in part attributed to larger sample size in western provinces than eastern provinces. Biotype 1 on both plants (i.e., wheat and barley), and biotype 2 on barley showed significant deviation from HWE (Table S4). Biotype 2 on wheat did not show significant departure from HWE.

### 3.2. Genetic Differentiation between S. avenae Biotypes

Based on pairwise *F*_ST_ values, high genetic divergence (*F*_ST_ > 0.15) between the two biotypes were detected in some cases, such as between AH1 (biotype 1 of Anhui) and GS2 (biotype 2 of Gansu), between HB1 (biotype 1 of Hubei) and GS2, between HN1 (biotype 1 of Henan) and GS2, between ZJ1 (biotype 1 of Zhejiang) and GS2, between GS1 (biotype 1 of Gansu) and SX2 (biotypes 2 of Shaanxi), and between XJ1 (biotype 1 of Xinjiang) and SX2 (Table 3). Indices of genetic differentiation (i.e., *F*_ST_) between the two biotypes in each province ranged from low to moderate (*F*_ST_ = 0.001–0.078). Moderate differentiation (0.05 < *F*_ST_ ≤ 0.15) between the two biotypes was found only in Shaanxi (*F*_ST_ = 0.078).

**Figure 3:**
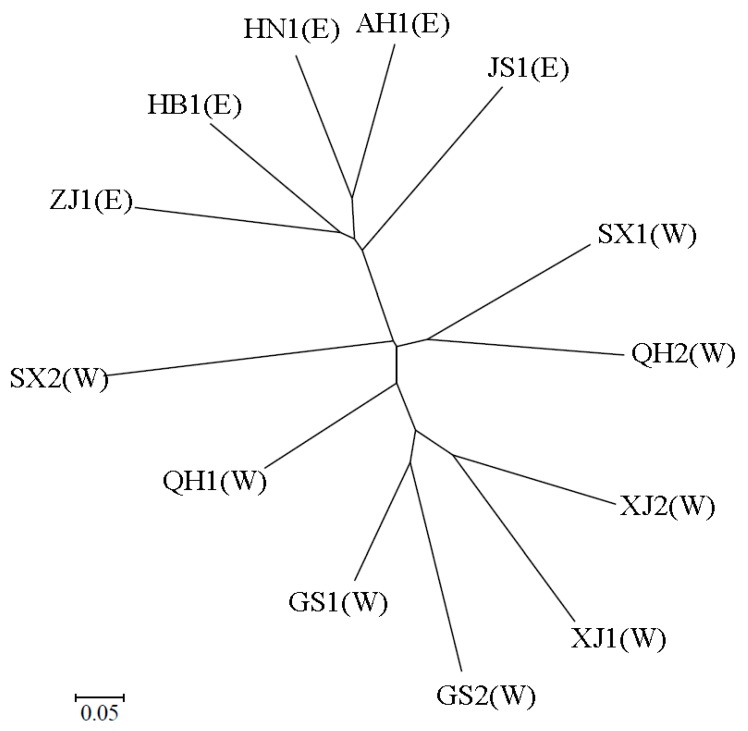
A neighbor-joining dendrogram of *Sitobion avenae* biotypes from nine provinces (AH1, biotype 1 of Anhui; HB1, biotype 1 of Hubei; HN1, biotype 1 of Henan; JS1, biotype 1 of Jiangsu; ZJ1, biotype 1 of Zhejiang; GS1 and GS2, biotypes 1 and 2 of Gansu; QH1 and QH2, biotypes 1 and 2 of Qinghai; SX1 and SX2, biotypes 1 and 2 of Shaanxi; XJ1 and XJ2, biotypes 1 and 2 of Xinjiang, respectively; E and W in brackets indicate eastern and western provinces, respectively).

A neighbor-joining tree based on Nei’s standard genetic distance (Ds) was created to show the genetic relationships of both *S. avenae* biotypes from nine locations (Figure 3). Clones of *S. avenae* formed four clades. One major clade consisted of biotype 1 of eastern provinces (i.e., Zhejiang, Hubei, Henan, Anhui, and Jiangsu). Another major clade included both biotypes from Xinjiang (XJ1 and XJ2) and Gansu (GS1 and GS2), as well as biotype 1 of Qinghai. Biotype 1 of Shaanxi and biotype 2 of Qinghai formed a third clade. Biotype 2 of Shaanxi alone formed a separate clade.

**Figure 4:**
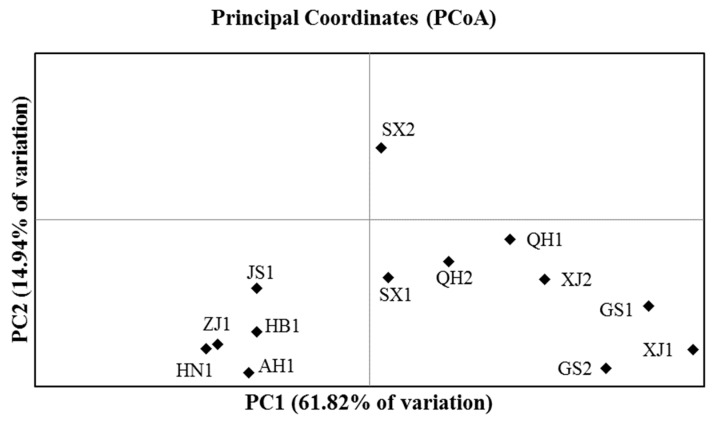
A plot of *Sitobion avenae* biotypes from nine provinces based on the principal coordinate analysis (PCoA) in GenAlEx (AH1, biotype 1 of Anhui; HB1, biotype 1 of Hubei; HN1, biotype 1 of Henan; JS1, biotype 1 of Jiangsu; ZJ1, biotype 1 of Zhejiang; GS1 and GS2, biotypes 1 and 2 of Gansu; QH1 and QH2, biotypes 1 and 2 of Qinghai; SX1 and SX2, biotypes 1 and 2 of Shaanxi; XJ1 and XJ2, biotypes 1 and 2 of Xinjiang, respectively).

Principal coordinate analyses (PCoA) showed that the first two axes accounted for 76.76% of the total variation (Figure 4; 61.82% and 14.94% for PC1 and PC2, respectively). The PCoA plot showed biotype 1 from eastern provinces of Zhejiang, Jiangsu, Anhui, Henan, and Hubei clustered in the lower left quadrant. Biotype 2 of Shaanxi (SX2) alone fell in the upper right quadrant. All other *S. avenae* clones fell in the lower right quadrant, and they included biotype 1 of Shaanxi (SX1), and both biotypes of Xinjiang (XJ1 and XJ2), Gansu (GS1 and GS2), and Qinghai (QH1 and QH2). 

**Figure 5:**
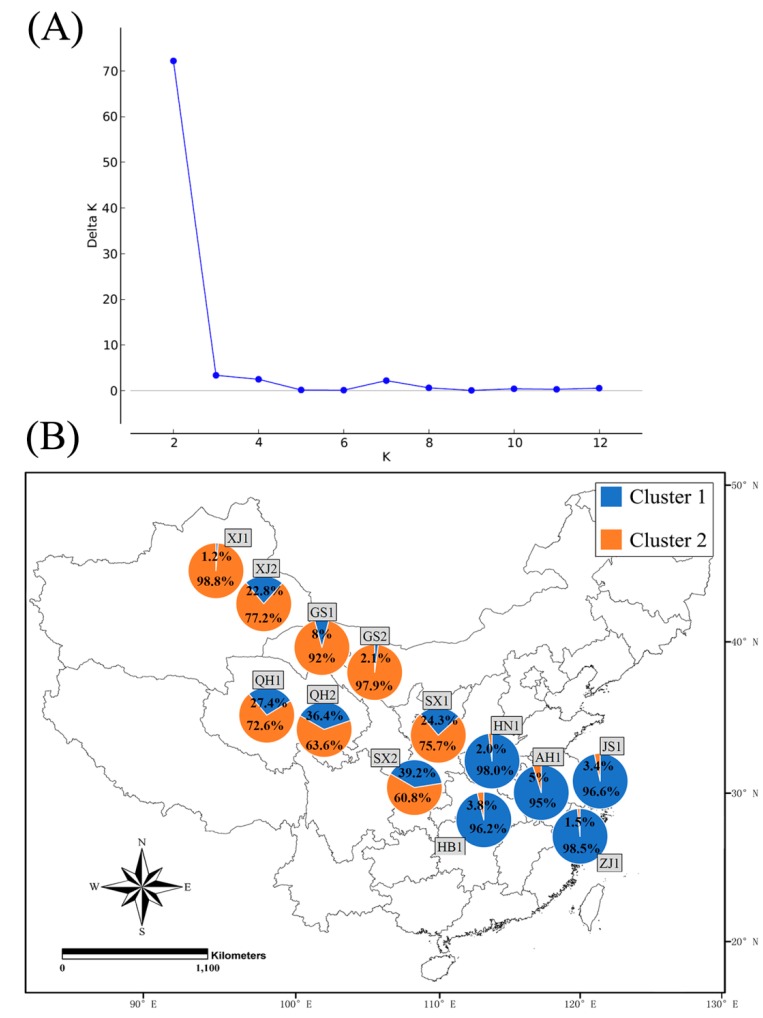
Clustering analyses of clones for two *Sitobion avenae* biotypes from nine provinces by using STRUCTURE (**A**: inference of the number of genetic clusters (K); **B**: the proportion of each cluster for both biotypes at each location; AH1, biotype 1 of Anhui; HB1, biotype 1 of Hubei; HN1, biotype 1 of Henan; JS1, biotype 1 of Jiangsu; ZJ1, biotype 1 of Zhejiang; GS1 and GS2, biotypes 1 and 2 of Gansu; QH1 and QH2, biotypes 1 and 2 of Qinghai; SX1 and SX2, biotypes 1 and 2 of Shaanxi; XJ1 and XJ2, biotypes 1 and 2 of Xinjiang, respectively; for more details, see Figure S1).

Based on the Bayesian analysis, the best supported model was K = 2 (Figure 5A), indicating that all clones of *S. avenae* in this study could be grouped into two genetic clusters. The majority of *S. avenae* clones for biotype 1 of Henan (98.0%), Hubei (96.2%), Anhui (95%), Jiangsu (96.6%) and Zhejiang (98.5%) belonged to cluster 1 (Figure 5B). On the contrary, most clones belonged to cluster 2 for western provinces with the proportion of these clones varying from 60.8% for biotype 2 of Shaanxi to 98.8% for biotype 1 of Xinjiang. For these western provinces, the genetic structure of biotype 1 clones seemed to be different from that of biotype 2, especially for Xinjiang (biotype 1: 1.2% cluster 1 and 98.8% cluster 2; biotype 2: 22.8% cluster 1 and 77.2% cluster 2) and Shaanxi (biotype 1: 24.3% cluster 1 and 75.7% cluster 2; biotype 2: 39.2% cluster 1 and 60.8% cluster 2).

**Figure 6:**
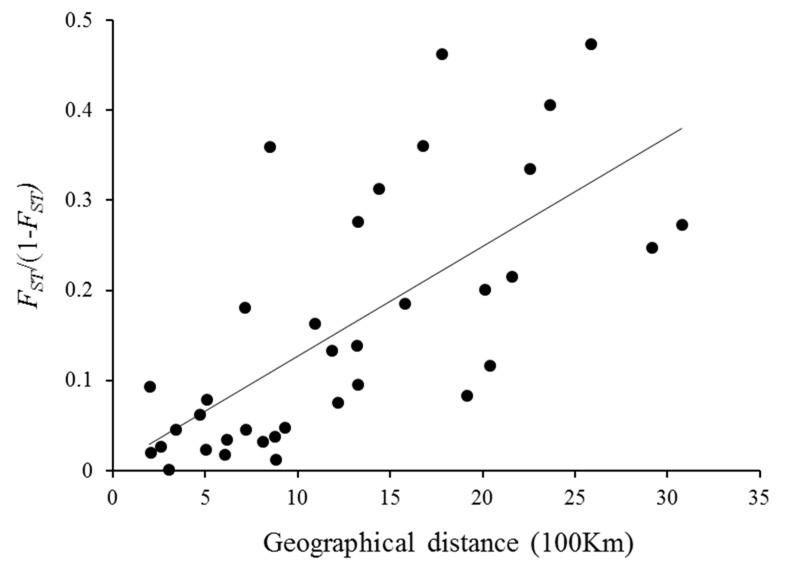
A scattered plot of genetic variation *F_ST_*/(1-*F_ST_*) vs. geographic distance for *Sitobion avenae* populations.

A significantly positive relationship was identified between genetic and geographic distances (Mantel test, r = 0.696, *p* < 0.001), indicating a significant effect of IBD (isolation-by-distance) among geographic populations of *S. avenae* (Figure 6).

AMOVA analyses showed that only 0.26% of the overall molecular variation was explained by variation between the two biotypes (Table 4; *p* = 0.822), showing no significant genetic differentiation between biotypes 1 and 2 of *S. avenae*. Another AMOVA analysis showed that 9.70% of the overall molecular variation could be explained by geographic location (*p* < 0.001), suggesting significant variation between *S. avenae* clones from eastern (i.e., Anhui, Hubei, Henan, Jiangsu, and Zhejiang) and western (i.e., Gansu, Qinghai, Shaanxi, and Xinjiang) provinces. AMOVA analyses of the host plant effect showed that it could contribute to only 0.48% (*p* = 0.950) of the overall molecular variation, suggesting that the overall variation between wheat and barley clones of *S. avenae* was non-significant in our sampling areas of this study. 

### 3.3. Gene Flow

Substantial gene flow (m) was found between the two biotypes (i.e., biotypes 1 and 2), as well as between eastern and western provinces (Table 5). In western provinces, the level of gene flow was found to be high (m = 0.2227) from biotype 1 to biotype 2, but it was non-significant in the reverse direction (i.e., from biotype 2 to biotype 1) (m = 0.0233). The migration of *S. avenae* toward eastern provinces was very low from both biotypes (biotype 1: m = 0.0053; biotype 2: m = 0.0487) of western provinces. Low rates of migration were also found from biotype 1 of eastern provinces to biotype 1 (m = 0.0258) and biotype 2 (m = 0.0823) of western provinces.

BayesAss analysis was also used to estimate gene flow between the two biotypes from barley and wheat (Table 6). Significant gene flow was identified between biotype 1 of barley and biotype 1 of wheat in both directions (from wheat to barley: 0.1784; from barley to wheat: 0.1205). Gene flow from biotype 1 of barley to biotype 2 of wheat was also significant (m = 0.1322), but it was negligible in the opposite direction (m = 0.0148). The level of gene flow from biotype 1 of wheat to biotype 2 of barley was relatively high (m = 0.2291). On wheat, significant gene flow was found from biotype 1 to biotype 2 (m = 0.1317), but not for the opposite direction. On barley, the level of gene flow between biotypes 1 and 2 was low in both directions (from biotype 1 to biotype 2: 0.0521; from biotype 2 to biotype 1: 0.0146). 

## 4. Discussion

### 4.1. Geographic Divergence among S. avenae Clones

Geographic structure and isolation of populations can play critical roles in the diversification of herbivorous insect populations. Despite that, a few studies have focused on the genetic differentiation of *S. avenae* clones in different areas of China [2,6,59], the relationship between geographic divergence and biotype development in *S. avenae* is still not well understood. In this study, in addition to differences in pairwise *F*_ST_ values, clones of *S. avenae* from western provinces had significantly higher gene diversity than those from eastern provinces, indicating differentiation between both areas. In addition, based on other genetic parameters like number of effective alleles (Ne), allelic richness (R), and Shannon’s information index (I), clones of western provinces for biotype 1 were also shown to have much higher genetic diversity than those of eastern provinces for the same biotype. It is generally believed that genetic differentiation between populations is low to negligible when *F*_ST_ ≤ 0.05, moderate when 0.05 < *F*_ST_ < 0.15, and high when *F*_ST_ ≥ 0.15 [2,59,60]. In this study, *S. avenae* clones from eastern provinces, which were all found to be biotype 1, showed little genetic divergence with pairwise *F*_ST_ values ranging from 0.010 to 0.054. However, some clones from western provinces showed high levels of genetic differentiation among them. For example, the pairwise *F*_ST_ value between XJ1 (biotype 1 of Xinjiang) and SX2 (biotype 2 of Shaanxi) was 0.221, and it was 0.186 between SX2 and GS2 (biotype 2 of Gansu). Clones from western provinces showed more scattered distribution in the PCoA plot than those from eastern provinces, suggesting higher levels of genetic divergence for clones from western provinces. Biotype 1 clones of both Xinjiang and Gansu showed high genetic differentiation from those of all five eastern provinces. A similar pattern was also found between GS2 and biotype 1 clones of eastern provinces. In addition to estimates of genetic diversity and distance (i.e., pairwise *F*_ST_), analyses of genetic structure of *S. avenae* clones and AMOVA (analyses of molecular variance) also indicated significant genetic divergence between eastern and western populations of this study. Significant geographic divergence of *S. avenae* populations could be attributed to limited long-distance dispersal from eastern to western provinces. Indeed, movements of biotype 1 of *S. avenae* were found to be very limited between eastern and western provinces in both directions. In China, the Qinling Mountains are clearly an important genetic barrier between populations to the east or west of the mountains [6]. The Mantel test in this study showed a significant positive relationship between genetic and geographic distances (r = 0.696, *p* < 0.001), indicating a significant isolation-by-distance (IBD) effect among different geographic populations of *S. avenae*. Our results indicate that geographic distance can play a significant role in the genetic structure of both *S. avenae* biotypes.

High genetic differentiation between *S. avenae* clones from eastern and western provinces can be closely related to geographic differences in their life cycles. In Europe, a majority of holocyclic clones of this aphid were discovered at locations with harsh winters (e.g., Romania), but more clones of obligate parthenogenesis were found in places with mild winters (e.g., southern France) [47,61]. According to the study of [62], overwintering with eggs and sexual reproduction of *S. avenae* in China could occur in western provinces, but probably not in all the eastern provinces of this study except Jiangsu. Thus, one possible explanation for high genetic differentiation between *S. avenae* clones from eastern and western provinces in China was that western provinces had lower winter temperatures, which could lead to more occurrences of sexual reproduction (recombination of alleles) for *S. avenae* in western provinces than in eastern provinces [63,64,65,66,67]. In this study, biotype 2 occurred in all four western provinces, instead of five eastern provinces. Thus, biotype 2 could be more likely to evolve from sexual lineages than asexual lineages. Differences in proportions of asexual and sexual lineages of *S. avenae* in both areas might contribute significantly to the abovementioned divergence and biotype development of this aphid. Sexual and asexual clones of *S. avenae* can be distinguished by rearing them under short day conditions in the lab [68]. Further studies in this respect can help to clarify the effects of reproductive mode on population differentiation of this aphid. Other geographic factors, such as selection from local natural enemies, adaptation to different environments (i.e., isolation by environment), and local crop domestication and agriculture, could also influence the divergence of *S. avenae* clones that we did not include in this study [69,70,71]. Further studies are needed to determine the effects of these specific geographic factors (e.g., relative proportions of sexual and asexual lineages of *S. avenae* in different areas) on the biotype development of *S. avenae*. 

### 4.2. Genetic Diversity and Divergence for S. avenae Biotypes

Host plant use and diet breadth are often assumed to be the critical factors influencing population divergence of insect herbivores. This is especially true for different aphid species, which are prone to develop variable host plant associated populations (e.g., biotypes) [17,20,33,34,35]. In our previous study, biotype 1 of the English grain aphid (*S. avenae*) was non-virulent on barley, whereas biotype 2 was characterized by its high virulence on barley (e.g., Zaoshu No.3) [22], clearly showing the potential effects of host plants on the divergence of *S. aveane* clones. In the present study, clones of biotype 1 in western provinces had higher genetic diversity than those of biotype 2 in the same area, based on estimates like number of effective alleles (Ne), allelic richness (R), and Shannon’s information index (I). When all samples were combined, *S. avenae* biotype 1 showed a higher Shannon’s information index (I) than biotype 2 (Figure 2B). In addition, in western provinces, the genetic structure of biotype 1 clones seemed to be different from that of biotype 2, especially for Xinjiang (biotype 1: 1.2% cluster 1 and 98.8% cluster 2; biotype 2: 22.8% cluster 1 and 77.2% cluster 2) and Shaanxi (biotype 1: 24.3% cluster 1 and 75.7% cluster 2; biotype 2: 39.2% cluster 1 and 60.8% cluster 2). These results indicated that there could be some degree of genetic divergence between both biotypes in relation to host plant use. 

However, the genetic divergence between the two biotypes (i.e., biotypes 1 and 2) of *S. avenae* was not significant in our analyses of molecular variance (AMOVA) with samples from nine provinces (Table 4). Genetic differentiation between barley and wheat clones of *S. avenae* in our sampling areas of nine provinces in China was not statistically significant either. Low genetic differentiation between both biotypes or between barley and wheat clones of *S. avenae* might be attributed to significant dispersal between these clones. For example, the levels of gene flow were found to be significant between biotype 1 of barley and biotype 1 of wheat in both directions (from wheat to barley: 0.1784; from barley to wheat: 0.1205). Similarly, substantial gene flow was also found between wheat and barley clones in our previous study [7]. Despite this, based on pairwise *F*_ST_ values and genetic structure analyses, significant levels of genetic divergence between barley and wheat clones were identified in Jiangsu and Zhejiang in our previous study [7]. This inconsistency may be due to different geographic scales used in both studies. Our previous study was limited to four eastern provinces (i.e., Jiangsu, Zhejiang, Hubei and Henan). Patterns of genetic divergence can strongly depend on the geographic scale being studied, because geography can strongly influence levels of gene flow in addition to environment and ecological contexts (i.e., host plant distribution) in a specific area [72]. Thus, geographic factors appeared to be more important than host plant use in the divergence of the two *S. avenae* biotypes. Some studies have proposed several mechanisms that may promote the maintenance of genetic structure of insect geographic populations and evolution of insect biotypes, such as differential composition of secondary endosymbionts, phenotypic plasticity, habitat isolation, and habitat persistence [2,21,24,30,31,73]. Future studies are needed to determine if these mechanisms can explain the development of biotypes in *S. avenae*. 

## 5. Conclusions

In summary, host plant use and geographic distance are some of the factors that can influence the genetic divergence of insect herbivores and development of insect biotypes. So far, the relative importance of both factors in the divergence of *S. avenae* clones is still not well understood. In this study, in terms of comparisons on genetic diversity between groups of *S. avenae* clones (e.g., eastern vs. western areas, and biotype 1 vs. biotype 2), the results of Nei’s gene diversity (Hs) were different from those of Shannon information index (I). The meaning of these differences is still difficult to interpret [59,74,75,76], which warrants further studies in the future. Surprisingly, we found that host-plant associated differentiation seemed to contribute little to the divergence of biotypes in *S. avenae*, since the plant factor contributed little to the total genetic differentiation of different clones of this aphid. In our previous study limited in eastern provinces, we found evidence for significant differentiation of barley and wheat clones of *S. avenae* [7]. Therefore, patterns of genetic divergence for this aphid can strongly depend on the geographic scale being studied. In addition, two biotypes of *S. avenae* showed significant structure and differentiation between eastern and western populations, and biotype 2 only occurred in western provinces. Therefore, it is very likely that the divergence of the two *S. avenae* biotypes [22] involved more geographic isolation and selection of some form than host plant affinity. The greater influence of geographic distance than host plant use on *S. avenae* genetic structure suggests that levels of host plant specialization in this aphid are still low, or that host shift is still recent in the evolutionary history, consistent with our previous life-history studies on this aphid [14,15]. Although host plant affinity is frequently identified to be the basis for evolution of biotypes, our data suggest that geographic factors, instead of host plant use, can be more important in biotype development in some insects (especially for those with wide-area distributions). Further studies are needed to identify key specific geographic elements involved in the process, and the underlying mechanisms. Genome scans, as well as candidate-gene studies, are also needed to identify which genes in insects have evolved in response to geographically variable selection. 

## Figures and Tables

**Table 1:** Collection information for *Sitobion avenae* samples.

Province	Code	Sample Size	Host	Coordinates	Collection Date
Zhejiang	ZJ	50	wheat, barley	120°54′ E; 30°52′ N	2016.04
Jiangsu	JS	64	wheat, barley	120°13′ E; 33°24′ N	2016.04
Anhui	AH	82	wheat, barley	116°53′ E; 33°59′ N	2016.04
Henan	HN	83	wheat, barley	114°02′ E; 33°00′ N	2016.04
Hubei	HB	105	wheat, barley	112°14′ E; 32°01′ N	2016.04
Gansu	GS	75	wheat, barley	100°46′ E; 38°38′ N	2016.06
Xinjaing	XJ	64	wheat, barley	92°53′ E; 43°36′ N	2016.06
Qinghai	QH	113	barley	101°44′ E; 36°43′ N	2016.07
Shaanxi	SX	117	wheat	108°05′ E; 34°17′ N	2016.04

**Table 2:** Genetic diversity indices of two *Sitobion avenae* biotypes from nine provinces.

Groups	N	Na	Ne	Hs	AR	I	Ho	He
**Western Provinces**								
**GS1**	34	7.833	3.483	0.732	4.010	1.404	0.466	0.645
**QH1**	49	11.333	4.336	0.763	4.851	1.779	0.573	0.753
**SX1**	10	7.000	4.841	0.801	5.091	1.639	0.617	0.752
**XJ1**	24	5.500	3.192	0.796	3.739	1.274	0.493	0.634
**Mean**	29.3	7.917	3.963	0.773	4.423	1.524	0.537	0.696
**GS2**	5	1.558	0.892	0.75	4.000	0.209	0.061	0.057
**QH2**	6	4.167	3.368	0.783	4.056	1.304	0.572	0.698
**SX2**	5	4.000	3.175	0.721	4.000	1.190	0.767	0.653
**XJ2**	5	4.833	3.973	0.746	4.833	1.350	0.733	0.670
**Mean**	5.3	3.640	2.852	0.750	4.222	1.013	0.533	0.520
**Eastern Provinces**								
**AH1**	8	5.333	3.614	0.651	4.450	1.370	0.646	0.668
**HB1**	21	8.500	4.301	0.761	4.733	1.655	0.587	0.739
**HN1**	13	6.167	3.128	0.678	4.121	1.360	0.731	0.654
**JS1**	12	8.000	4.657	0.717	5.259	1.711	0.514	0.751
**ZJ1**	10	5.333	3.812	0.658	4.267	1.378	0.592	0.687
**Mean**	12.8	6.667	3.902	0.693	4.566	1.495	0.614	0.700

Note: AH1, biotype 1 of Anhui; HB1, biotype 1 of Hubei; HN1, biotype 1 of Henan; JS1, biotype 1 of Jiangsu; ZJ1, biotype 1 of Zhejiang; GS1 and GS2, biotypes 1 and 2 of Gansu, respectively; QH1 and QH2, biotypes 1 and 2 of Qinghai; SX1 and SX2, biotypes 1 and 2 of Shaanxi; XJ1 and XJ2, biotypes 1 and 2 of Xinjiang; Na, number of alleles; Ne, number of effective alleles; Hs, gene diversity; AR, allelic richness; I, Shannon’s information index; Ho, observed heterozygosity; He, expected heterozygosity.

**Table 3:** Pairwise genetic distance estimates (*F*_ST_) for two *Sitobion avenae* biotypes from nine provinces.

	AH1	HB1	HN1	JS1	ZJ1	GS1	QH1	SX1	XJ1	GS2	QH2	SX2	XJ2
**AH1**													
**HB1**	0.014												
**HN1**	0.001	0.019											
**JS1**	0.003	0.013	0.014										
**ZJ1**	0.054	0.012	0.052	0.020									
**GS1**	**0.174**	**0.164**	**0.195**	**0.170**	**0.213**								
**QH1**	0.103	0.093	0.125	0.091	0.121	0.055							
**SX1**	0.037	0.046	0.057	0.038	0.091	0.117	0.064						
**XJ1**	**0.211**	**0.191**	**0.244**	**0.197**	**0.241**	0.029	0.095	0.140					
**GS2**	**0.152**	**0.151**	**0.188**	0.129	**0.190**	0.031	0.051	0.106	0.067				
**QH2**	0.071	0.061	0.090	0.059	0.112	0.076	0.001	0.012	0.123	0.051			
**SX2**	0.116	0.092	0.117	0.082	0.130	**0.175**	0.088	0.078	**0.221**	**0.186**	0.090		
**XJ2**	0.117	0.098	0.139	0.104	0.145	0.047	0.040	0.053	0.041	0.089	0.061	0.120	

Note: AH1, biotype 1 of Anhui; HB1, biotype 1 of Hubei; HN1, biotype 1 of Henan; JS1, biotype 1 of Jiangsu; ZJ1, biotype 1 of Zhejiang; GS1 and GS2, biotypes 1 and 2 of Gansu, respectively; QH1 and QH2, biotypes 1 and 2 of Qinghai; SX1 and SX2, biotypes 1 and 2 of Shaanxi; XJ1 and XJ2, biotypes 1 and 2 of Xinjiang, respectively; high levels of genetic divergence (*F*_ST_ > 0.15) highlighted in bold.

**Table 4:** Analyses of molecular variance (AMOVA) for effects of biotype, geography and plant in the divergence of *Sitobion avenae* clones.

Sources of Variation	d. f.	Sum of Squares	Variance Components	Percentage of Variation	*p*-Value
**Biotype effect**					
Among groups	1	2.51	0.05 Va	0.26	0.822
Among populations within groups	11	110	0.25 Vb	11.56	**<0.001**
Within populations	391	839.32	2.15 Vc	88.18	**<0.001**
**Geographic effect**					
Among groups	1	49.76	0.24 Va	9.70	**<0.001**
Among populations within groups	11	62.74	0.13 Vb	4.98	**<0.001**
Within populations	391	839.32	2.15 Vc	85.32	**<0.001**
**Plant effect**					
Among groups	1	4.616	0.05 Va	0.48	0.950
Among populations within groups	14	117.99	0.28 Vb	12.40	**<0.001**
Within populations	388	829.21	2.14 Vc	87.12	**<0.001**

Note: Clones of *S. avenae* were divided into two groups in terms of biotype [group 1 (i.e., AH1,GS1, HB1, HN1, JS1, QH1, SX1, XJ1, ZJ1) and group 2 (i.e., GS2, QH2, SX2, XJ2)], two geographic groups [group 1 (i.e., eastern provinces: Anhui, Hubei, Henan, Jiangsu, Zhejiang) and group 2 (i.e., western provinces: Gansu, Qinghai, Shaanxi, Xinjiang)], and two plant-associated groups (group 1, clones on wheat; group 2, clones on barley); significant effects highlighted in bold.

**Table 5:** Estimates of gene flow between *Sitobion avenae* biotypes of eastern and western provinces inferred from BayesAss analyses.

Population	E1	W1	W2
E1		0.0258 (0.0102–0.0463)	0.0823 (0.0339–0.1431)
W1	0.0053 (0.0001–0.0177)		0.2227 (0.1568–0.2804)
W2	0.0487 (0.0190–0.0859)	0.0233 (0.0107–0.0407)	

Note: 95% confidence intervals presented in parentheses; direction of gene flow is from populations in the left column to those along the top row; E1, biotype 1 of eastern provinces (i.e., Anhui, Hubei, Henan, Jiangsu, and Zhejiang); W1, biotype 1 of western provinces (i.e., Gansu, Qinghai, Shaanxi, and Xinjiang); W2, biotype 2 of western provinces.

**Table 6:** Estimates of gene flow between *Sitobion avenae* biotypes collected on barley and wheat inferred from BayesAss analyses.

Population	BA1	BA2	WH1	WH2
BA1		0.0521 (0.0057–0.1265)	0.1205 (0.0833–0.1613)	0.1322 (0.0429–0.2421)
BA2	0.0246 (0.0058–0.0519)		0.0173 (0.0042–0.0397)	0.0295 (0.0004–0.1066)
WH1	0.1784 (0.1410–0.2140)	0.2291 (0.1477–0.3003)		0.1317 (0.0426–0.2348)
WH2	0.0148 (0.0006–0.0372)	0.0128 (0.0001–0.05513)	0.0031 (0.0001–0.0127)	

Note: 95% confidence intervals presented in parentheses; direction of gene flow is from populations in the left column to those along the top row; BA1, biotype 1 on barley; BA2, biotype 2 on barley; WH1, biotype 1 on wheat; WH2, biotype 2 on wheat.

## Supplementary Materials

The following are available online at https://www.mdpi.com/2075-4450/11/2/117/s1.
