# Supplementary material for: Genetic Divergence of Two Sitobion avenae Biotypes on Barley and Wheat in China
Source: Insects. 2020 Feb 11;11(2):117. doi: 10.3390/insects11020117 (PMC7073604; doi:10.3390/insects11020117)

Table S1. Primer sequences of six microsatellite loci used for genotyping individuals of *Sitobion avenae*

Locus	Forward primer (5' → 3')	Reverse primer (5' → 3')	References
Sm10	TCTGCTGCATTACTGTTGGC	TCGTCTACTTCGCCGTCA	Simon et al. 1999
S5.L	GGACGACTCGTTAGTATAGGTGG	CTATCTCTACCGTTTCGAATCG	Wilson et al. 2004
Sa4Σ	GTGACGTATACGCGATGCG	GACGTCGATATTAGCCTAGCC	Simon et al. 1999
S17b	TTCTGGCTTCATTCCGGTCG	CGTCGCGTTAGTGAACCGTG	Wilson et al. 2004
Sm17	TGGACATTTCATCGTTCGC	ATGCGTTCGAGTTTACCTGC	Simon et al. 1999
Sm12	GATCCCCCTCAAATCACTCA	GTACTCCCAACCTCTGATGAGC	Simon et al. 1999

Table S2. List of multilocus genotypes for *Sitobion avenae* biotypes 1 and 2 collected from nine provinces of China (six microsatellite loci used in genotyping)

Genotype	Biotype	Province	Host	Locus											
				Sm10		S5.L		Sa4Σ		Sm17b		Sm17		Sm12	
AH1-01	1	Anhui	Barley	164	166	225	227	150	150	196	196	94	98	166	166
AH1-02	1	Anhui	Barley	156	164	223	226	163	163	196	196	94	100	151	158
AH1-03	1	Anhui	Barley	156	164	214	223	163	163	196	196	94	98	149	158
AH1-04	1	Anhui	Barley	156	164	223	226	163	163	196	196	94	98	133	158
AH1-05	1	Anhui	Wheat	164	166	214	223	157	163	196	196	94	94	151	166
AH1-06	1	Anhui	Wheat	151	154	231	234	157	157	192	192	94	94	155	166
AH1-07	1	Anhui	Wheat	151	154	214	227	161	165	194	198	100	100	135	149
AH1-08	1	Anhui	Wheat	156	164	216	223	163	163	196	196	94	100	149	158
GS1-01	1	Gansu	Barley	191	191	227	227	163	165	202	202	94	98	149	149
GS1-02	1	Gansu	Barley	154	164	227	227	152	165	202	202	94	94	145	158
GS1-03	1	Gansu	Barley	164	164	227	227	152	165	202	204	94	94	160	160
GS1-04	1	Gansu	Barley	164	164	227	227	165	165	202	202	94	94	156	156
GS1-05	1	Gansu	Barley	156	164	228	230	165	165	202	202	94	98	156	156
GS1-06	1	Gansu	Barley	164	164	223	225	165	165	194	209	94	100	145	158
GS1-07	1	Gansu	Barley	164	164	223	225	165	165	202	202	94	94	147	147
GS1-08	1	Gansu	Barley	156	156	223	225	165	165	202	202	98	98	149	154
GS1-09	1	Gansu	Barley	164	164	223	228	165	165	192	192	94	98	154	156
GS1-10	1	Gansu	Barley	156	156	222	227	156	163	202	202	94	94	147	147
GS1-11	1	Gansu	Barley	156	164	223	227	165	165	192	192	94	98	149	158
GS1-12	1	Gansu	Barley	164	164	223	227	165	165	202	204	94	100	164	164
GS1-13	1	Gansu	Barley	156	162	227	227	152	157	202	204	94	94	139	139
GS1-14	1	Gansu	Barley	164	164	227	227	152	165	202	202	94	100	149	156
GS1-15	1	Gansu	Wheat	154	164	212	227	152	157	202	202	94	94	128	128
GS1-16	1	Gansu	Wheat	154	156	223	223	152	152	204	204	94	94	128	147
GS1-17	1	Gansu	Wheat	154	156	224	227	152	169	202	202	94	94	130	130
GS1-18	1	Gansu	Wheat	154	166	227	227	165	167	204	204	94	94	149	156
GS1-19	1	Gansu	Wheat	154	164	212	227	157	157	204	204	94	94	135	156
GS1-20	1	Gansu	Wheat	164	164	223	223	161	163	196	202	94	98	149	156
GS1-21	1	Gansu	Wheat	164	164	227	227	165	167	194	204	94	100	149	156
GS1-22	1	Gansu	Wheat	156	164	227	227	165	167	202	202	94	94	139	149
GS1-23	1	Gansu	Wheat	164	164	225	227	165	167	196	204	94	94	128	139
GS1-24	1	Gansu	Wheat	164	164	227	227	152	165	204	204	94	94	128	147
GS1-25	1	Gansu	Wheat	164	164	227	227	165	167	202	202	94	94	139	149
GS1-26	1	Gansu	Wheat	156	164	223	225	156	163	202	202	94	94	149	149
GS1-27	1	Gansu	Wheat	156	164	227	227	152	167	204	204	94	98	151	156
GS1-28	1	Gansu	Wheat	156	156	227	227	157	165	194	204	94	100	135	156
GS1-29	1	Gansu	Wheat	164	164	223	227	152	167	204	204	94	100	147	147
GS1-30	1	Gansu	Wheat	154	164	216	227	152	165	202	202	94	98	149	149

GS1-31	1	Gansu	Wheat	164	164	227	227	165	165	202	202	98	98	138	138
GS1-32	1	Gansu	Wheat	154	166	227	227	165	167	204	204	94	98	139	139
GS1-33	1	Gansu	Wheat	154	154	216	227	157	163	202	202	94	94	149	158
GS1-34	1	Gansu	Wheat	164	164	211	225	152	157	204	204	98	98	128	149
GS2-01	2	Gansu	Barley	154	162	227	227	152	169	202	202	94	94	130	130
GS2-02	2	Gansu	Barley	164	164	223	227	157	157	202	202	94	98	149	158
GS2-03	2	Gansu	Barley	154	162	216	227	167	167	206	206	94	100	139	139
GS2-04	2	Gansu	Barley	156	164	223	227	152	157	192	192	94	100	149	156
GS2-05	2	Gansu	Wheat	154	162	227	227	152	167	204	204	94	94	147	147
HB1-01	1	Hubei	Barley	155	164	223	226	163	163	196	196	94	102	151	158
HB1-02	1	Hubei	Barley	154	154	217	226	163	165	194	194	98	100	148	148
HB1-03	1	Hubei	Barley	164	164	223	226	163	163	196	196	94	100	135	158
HB1-04	1	Hubei	Barley	154	157	228	228	173	175	198	198	102	108	140	140
HB1-05	1	Hubei	Barley	150	158	227	227	153	156	196	196	102	102	187	187
HB1-06	1	Hubei	Barley	155	164	216	223	163	163	196	196	94	100	149	158
HB1-07	1	Hubei	Barley	154	164	218	227	159	165	194	194	100	100	148	149
HB1-08	1	Hubei	Barley	155	164	223	223	163	163	196	202	94	98	155	158
HB1-09	1	Hubei	Barley	155	164	226	226	163	165	196	202	94	98	149	158
HB1-10	1	Hubei	Barley	154	154	217	223	169	165	196	196	98	100	155	155
HB1-11	1	Hubei	Barley	154	154	217	223	160	165	196	196	98	100	155	155
HB1-12	1	Hubei	Barley	154	164	223	223	161	165	196	196	94	100	135	149
HB1-13	1	Hubei	Barley	154	166	222	222	171	171	222	222	100	102	187	187
HB1-14	1	Hubei	Wheat	164	166	225	227	157	163	196	196	94	94	151	166
HB1-15	1	Hubei	Wheat	156	164	223	223	163	163	194	194	94	98	155	158
HB1-16	1	Hubei	Wheat	156	164	216	223	163	163	196	196	94	100	149	155
HB1-17	1	Hubei	Wheat	179	179	223	227	165	169	194	194	98	100	155	155
HB1-18	1	Hubei	Wheat	154	164	223	226	163	165	194	194	94	98	149	158
HB1-19	1	Hubei	Wheat	156	164	223	226	163	163	196	196	94	102	151	158
HB1-20	1	Hubei	Wheat	156	156	214	227	163	165	196	196	98	100	139	155
HB1-21	1	Hubei	Wheat	154	154	214	227	163	165	196	196	100	100	135	149
HN1-01	1	Henan	Barley	156	164	217	223	163	163	196	196	94	98	149	158
HN1-02	1	Henan	Barley	156	164	216	223	163	163	196	196	94	98	149	158
HN1-03	1	Henan	Barley	156	164	214	223	163	163	196	196	94	100	149	158
HN1-04	1	Henan	Barley	156	164	214	223	163	165	194	196	94	98	149	158
HN1-05	1	Henan	Barley	154	164	216	223	163	163	196	196	94	100	149	158
HN1-06	1	Henan	Barley	151	154	214	223	161	165	194	196	100	100	135	149
HN1-07	1	Henan	Barley	164	166	214	223	163	163	194	196	94	98	135	158
HN1-08	1	Henan	Barley	154	173	223	229	155	182	206	206	102	106	123	123
HN1-09	1	Henan	Barley	154	156	216	229	175	175	165	169	94	100	135	200
HN1-10	1	Henan	Wheat	156	164	214	223	163	163	194	196	94	98	149	158
HN1-11	1	Henan	Wheat	164	164	223	226	163	163	194	196	94	100	135	158
HN1-12	1	Henan	Wheat	156	164	217	223	163	163	196	196	94	100	149	158
HN1-13	1	Henan	Wheat	164	166	225	227	157	163	196	196	94	94	166	166

JS1-01	1	Jiangsu	Barley	147	160	223	225	163	163	197	197	94	94	208	208
JS1-02	1	Jiangsu	Barley	162	165	223	225	157	161	240	240	94	108	163	163
JS1-03	1	Jiangsu	Barley	179	188	223	227	165	169	196	196	98	100	155	155
JS1-04	1	Jiangsu	Barley	156	156	225	225	159	165	216	216	96	100	174	174
JS1-05	1	Jiangsu	Barley	156	156	216	223	163	163	197	197	94	100	149	158
JS1-06	1	Jiangsu	Barley	154	166	226	229	152	167	196	205	94	102	166	166
JS1-07	1	Jiangsu	Barley	154	154	215	230	163	163	196	196	100	100	147	147
JS1-08	1	Jiangsu	Barley	156	164	216	227	163	163	196	196	94	100	149	158
JS1-09	1	Jiangsu	Wheat	154	154	215	230	163	163	194	194	100	100	147	147
JS1-10	1	Jiangsu	Wheat	156	164	216	223	163	163	196	196	94	100	150	158
JS1-11	1	Jiangsu	Wheat	156	164	223	227	163	163	196	196	94	98	155	158
JS1-12	1	Jiangsu	Wheat	164	164	216	223	164	164	196	196	94	98	150	158
QH1-01	1	Qinghai	Barley	154	156	214	227	161	165	194	194	100	100	135	155
QH1-02	1	Qinghai	Barley	151	177	207	208	159	163	184	184	96	96	178	178
QH1-03	1	Qinghai	Barley	154	156	214	224	163	165	194	194	94	98	135	155
QH1-04	1	Qinghai	Barley	179	179	223	227	165	169	194	205	98	100	156	156
QH1-05	1	Qinghai	Barley	154	170	223	237	151	151	196	196	94	104	158	158
QH1-06	1	Qinghai	Barley	156	156	227	227	152	169	204	204	94	100	149	155
QH1-07	1	Qinghai	Barley	154	166	224	226	163	167	204	204	94	100	155	155
QH1-08	1	Qinghai	Barley	164	164	227	227	163	165	204	204	94	100	139	145
QH1-09	1	Qinghai	Barley	164	164	227	227	157	163	206	210	94	100	149	155
QH1-10	1	Qinghai	Barley	164	164	226	228	154	165	196	196	94	98	139	145
QH1-11	1	Qinghai	Barley	156	156	227	227	154	169	204	204	94	100	149	155
QH1-12	1	Qinghai	Barley	154	164	223	226	157	165	204	204	94	100	148	155
QH1-13	1	Qinghai	Barley	150	154	223	225	159	163	208	208	98	98	0	0
QH1-14	1	Qinghai	Barley	156	164	227	227	157	167	196	196	94	98	149	155
QH1-15	1	Qinghai	Barley	156	156	224	226	157	165	204	204	94	94	149	155
QH1-16	1	Qinghai	Barley	164	164	223	223	157	163	208	208	94	94	155	155
QH1-17	1	Qinghai	Barley	154	156	227	227	152	169	204	204	94	100	149	155
QH1-18	1	Qinghai	Barley	164	164	223	223	163	165	206	208	94	98	155	155
QH1-19	1	Qinghai	Barley	164	164	227	227	163	165	206	206	94	94	149	155
QH1-20	1	Qinghai	Barley	154	166	223	225	163	165	204	204	94	94	149	155
QH1-21	1	Qinghai	Barley	156	156	227	227	161	163	194	194	94	98	156	156
QH1-22	1	Qinghai	Barley	156	156	223	227	157	163	202	204	100	100	156	156
QH1-23	1	Qinghai	Barley	154	164	213	227	163	165	200	206	98	102	149	155
QH1-24	1	Qinghai	Barley	154	156	223	225	157	165	204	204	98	102	149	155
QH1-25	1	Qinghai	Barley	154	164	213	227	163	165	200	202	98	100	139	145
QH1-26	1	Qinghai	Barley	164	164	223	223	152	157	194	204	94	94	149	155
QH1-27	1	Qinghai	Barley	154	164	212	226	163	165	204	204	94	94	156	156
QH1-28	1	Qinghai	Barley	154	164	227	227	163	165	204	204	94	100	149	155
QH1-29	1	Qinghai	Barley	156	166	227	227	157	165	204	204	94	94	149	155
QH1-30	1	Qinghai	Barley	154	156	227	227	161	163	194	194	94	98	156	156
QH1-31	1	Qinghai	Barley	164	164	227	227	152	163	204	204	94	94	139	145

QH1-32	1	Qinghai	Barley	164	164	215	227	152	165	204	204	94	94	147	154
QH1-33	1	Qinghai	Barley	154	164	227	227	157	165	204	204	98	102	147	154
QH1-34	1	Qinghai	Barley	156	186	225	227	157	165	194	204	94	98	156	156
QH1-35	1	Qinghai	Barley	154	164	227	227	157	165	206	206	94	94	139	139
QH1-36	1	Qinghai	Barley	156	164	227	227	157	165	204	204	94	94	156	156
QH1-37	1	Qinghai	Barley	156	164	213	227	157	165	210	210	94	94	156	156
QH1-38	1	Qinghai	Barley	164	164	214	225	163	168	202	206	94	94	139	139
QH1-39	1	Qinghai	Barley	154	156	223	227	157	163	202	204	94	94	149	155
QH1-40	1	Qinghai	Barley	156	156	213	213	163	165	204	204	94	94	149	155
QH1-41	1	Qinghai	Barley	154	164	227	241	163	171	204	204	94	94	156	156
QH1-42	1	Qinghai	Barley	160	174	225	225	152	157	243	243	96	96	158	158
QH1-43	1	Qinghai	Barley	156	164	227	241	163	165	194	194	96	96	139	139
QH1-44	1	Qinghai	Barley	164	164	227	241	157	157	204	204	96	96	147	154
QH1-45	1	Qinghai	Barley	151	177	206	208	159	163	184	184	98	100	150	158
QH1-46	1	Qinghai	Barley	154	156	223	227	152	165	204	204	94	98	139	139
QH1-47	1	Qinghai	Barley	164	164	225	228	157	157	204	204	94	100	147	154
QH1-48	1	Qinghai	Barley	151	177	206	208	159	163	184	184	94	100	150	158
QH1-49	1	Qinghai	Barley	154	164	227	227	165	167	204	204	94	94	149	155
QH2-01	2	Qinghai	Barley	156	164	223	227	163	163	196	196	94	98	156	156
QH2-02	2	Qinghai	Barley	158	167	222	222	164	164	200	200	94	100	0	0
QH2-03	2	Qinghai	Barley	154	154	223	227	157	163	194	204	94	102	149	155
QH2-04	2	Qinghai	Barley	164	164	227	227	157	157	204	204	98	102	156	156
QH2-05	2	Qinghai	Barley	156	164	213	227	163	165	206	206	94	100	149	155
QH2-06	2	Qinghai	Barley	156	164	213	227	163	165	206	206	94	94	149	155
SX1-01	1	Shaanxi	Wheat	164	164	223	223	163	163	194	196	94	100	135	157
SX1-02	1	Shaanxi	Wheat	156	164	223	227	163	165	196	196	96	104	150	157
SX1-03	1	Shaanxi	Wheat	154	164	214	223	163	165	196	196	94	98	159	159
SX1-04	1	Shaanxi	Wheat	164	164	213	223	163	165	194	196	94	100	156	158
SX1-05	1	Shaanxi	Wheat	164	166	225	227	157	163	196	196	94	94	165	174
SX1-06	1	Shaanxi	Wheat	164	164	225	227	167	169	200	200	94	94	139	139
SX1-07	1	Shaanxi	Wheat	158	167	222	222	164	164	198	200	94	98	135	144
SX1-08	1	Shaanxi	Wheat	158	167	222	226	164	164	198	200	94	98	142	144
SX1-09	1	Shaanxi	Wheat	158	167	222	222	164	164	200	200	94	100	135	144
SX1-10	1	Shaanxi	Wheat	156	156	227	227	163	165	204	204	94	100	149	155
SX2-01	2	Shaanxi	Wheat	156	164	225	227	163	163	194	196	96	102	135	157
SX2-02	2	Shaanxi	Wheat	154	164	217	223	157	163	194	196	96	104	150	157
SX2-03	2	Shaanxi	Wheat	156	156	223	227	165	165	204	204	96	104	141	147
SX2-04	2	Shaanxi	Wheat	156	164	223	223	163	165	196	196	96	104	150	157
SX2-05	2	Shaanxi	Wheat	156	164	224	227	163	165	204	204	96	104	139	149
XJ1-01	1	Xinjiang	Barley	154	164	227	227	165	167	192	192	94	94	155	155
XJ1-02	1	Xinjiang	Barley	164	164	227	227	157	165	200	204	98	98	160	160
XJ1-03	1	Xinjiang	Barley	164	164	225	227	157	157	200	200	98	98	141	155
XJ1-04	1	Xinjiang	Barley	164	166	225	227	153	167	200	200	98	98	160	160

XJ1-05	1	Xinjiang	Wheat	164	166	226	228	157	165	202	204	94	98	139	156
XJ1-06	1	Xinjiang	Wheat	164	170	226	228	152	165	202	202	94	94	147	154
XJ1-07	1	Xinjiang	Wheat	164	166	225	227	165	165	202	202	94	94	174	174
XJ1-08	1	Xinjiang	Wheat	154	166	214	225	165	165	204	204	94	98	141	141
XJ1-09	1	Xinjiang	Wheat	191	191	223	227	157	165	202	202	94	98	147	147
XJ1-10	1	Xinjiang	Wheat	164	170	218	227	165	165	202	202	94	98	139	147
XJ1-11	1	Xinjiang	Wheat	154	164	223	227	165	165	202	202	94	94	139	156
XJ1-12	1	Xinjiang	Wheat	164	164	225	227	165	165	202	202	98	98	147	147
XJ1-13	1	Xinjiang	Wheat	164	170	225	227	165	165	202	202	94	94	160	160
XJ1-14	1	Xinjiang	Wheat	164	170	225	227	157	165	202	202	94	94	154	156
XJ1-15	1	Xinjiang	Wheat	164	164	227	227	152	157	200	200	98	98	139	147
XJ1-16	1	Xinjiang	Wheat	164	170	225	227	157	167	202	202	94	94	139	147
XJ1-17	1	Xinjiang	Wheat	164	170	227	227	152	165	202	202	94	94	147	147
XJ1-18	1	Xinjiang	Wheat	154	164	214	225	152	165	202	202	94	94	139	139
XJ1-19	1	Xinjiang	Wheat	164	170	223	227	152	157	202	202	94	94	139	156
XJ1-20	1	Xinjiang	Wheat	164	170	225	227	152	165	202	202	94	94	139	139
XJ1-21	1	Xinjiang	Wheat	154	166	227	227	152	165	202	202	94	94	154	156
XJ1-22	1	Xinjiang	Wheat	154	154	225	227	165	167	202	202	94	94	155	155
XJ1-23	1	Xinjiang	Wheat	154	154	225	227	152	165	204	204	94	94	154	156
XJ1-24	1	Xinjiang	Wheat	164	170	224	227	163	165	204	204	94	98	147	147
XJ2-01	2	Xinjiang	Barley	154	164	214	223	163	165	194	196	94	100	147	157
XJ2-02	2	Xinjiang	Barley	164	170	214	225	165	165	204	204	94	94	139	154
XJ2-03	2	Xinjiang	Barley	164	170	226	228	165	165	206	208	94	98	174	174
XJ2-04	2	Xinjiang	Barley	164	170	223	227	152	165	202	202	94	94	154	156
XJ2-05	2	Xinjiang	Barley	154	164	224	227	163	165	204	208	94	100	160	160
ZJ1-01	1	Zhejiang	Barley	154	156	217	223	159	165	196	196	98	100	156	156
ZJ1-02	1	Zhejiang	Barley	156	164	217	223	163	163	196	196	94	100	150	158
ZJ1-03	1	Zhejiang	Barley	154	166	214	221	155	155	196	196	98	98	0	0
ZJ1-04	1	Zhejiang	Barley	154	156	216	227	155	157	222	222	94	98	0	0
ZJ1-05	1	Zhejiang	Wheat	154	156	218	223	159	165	196	196	98	100	156	156
ZJ1-06	1	Zhejiang	Wheat	154	156	218	223	159	165	196	196	98	100	155	165
ZJ1-07	1	Zhejiang	Wheat	154	154	226	226	163	163	194	194	94	100	148	158
ZJ1-08	1	Zhejiang	Wheat	154	154	215	230	163	163	194	194	100	100	148	158
ZJ1-09	1	Zhejiang	Wheat	156	164	218	223	163	163	196	196	94	100	149	158
ZJ1-10	1	Zhejiang	Wheat	154	154	215	230	163	165	194	194	100	100	148	158

Note: 0, indicates the loss of microsatellite alleles.

Table S3. Testing for Hardy-Weinberg equilibrium for different geographic populations of *Sitobion avenae*

	Locus	Geographic population								
		Gansu	Qinghai	Shaanxi	Xinjiang	Anhui	Hubei	Henan	Jiangsu	Zhejiang
HWE-*P*	All loci	< 0.001	< 0.001	0.0025	< 0.001	0.0793	< 0.001	0.0788	< 0.001	0.0091
	SM10	0.0020	0.0051	0.3265	0.0385	0.1407	0.0102	0.0679	0.0098	0.3125
	S5.L	0.3279	0.0016	0.0383	0.0467	0.3452	< 0.001	0.0196	0.4571	0.2674
	Sa4Σ	0.1181	0.0005	0.0579	0.3797	0.0026	0.0122	0.0118	0.0019	0.1098
	SM17b	< 0.001	< 0.001	0.0091	< 0.001	0.0051	< 0.001	0.0338	< 0.001	0.0002
	SM17	0.2857	0.0029	0.2402	0.0309	0.4965	0.3866	0.1649	0.5442	0.4767
	SM12	< 0.001	< 0.001	0.0455	< 0.001	0.5912	0.0006	0.0202	< 0.001	0.1712

Note: HWE-*P*, *P* value for testing Hardy-Weinberg equilibrium.

Table S4. Testing for Hardy-Weinberg equilibrium for two *Sitobion avenae* biotypes on wheat and barley

	Locus	Wheat		Barley	
		Biotype 1	Biotype 2	Biotype 1	Biotype 2
HWE-*P*	All loci	< 0.001	0.1328	< 0.001	< 0.001
	SM10	< 0.001	0.4156	< 0.001	0.4908
	S5.L	0.0446	0.4425	< 0.001	0.1042
	Sa4Σ	0.0022	0.4373	< 0.001	0.0004
	SM17b	< 0.001	0.0996	< 0.001	< 0.001
	SM17	0.0175	0.2035	0.0016	0.1401
	SM12	< 0.001	0.4805	< 0.001	< 0.001

Note: HWE-*P*, *P* value for testing Hardy-Weinberg equilibrium.

Figure S1. Bar plot for clustering analyses of clones for two *Sitobion avenae* biotypes from nine provinces by using STRUCTURE (all individuals assigned to two clusters; each individual represented by a vertical bar; percentages in the plot showing the proportion of cluster 1 in each group; AH1, biotype 1 of Anhui; HB1, biotype 1 of Hubei; HN1, biotype 1 of Henan; JS1, biotype 1 of Jiangsu; ZJ1, biotype 1 of Zhejiang; GS1 and GS2, biotypes 1 and 2 of Gansu; QH1 and QH2, biotypes 1 and 2 of Qinghai; SX1 and SX2, biotypes 1 and 2 of Shaanxi; XJ1 and XJ2, biotypes 1 and 2 of Xinjiang, respectively)

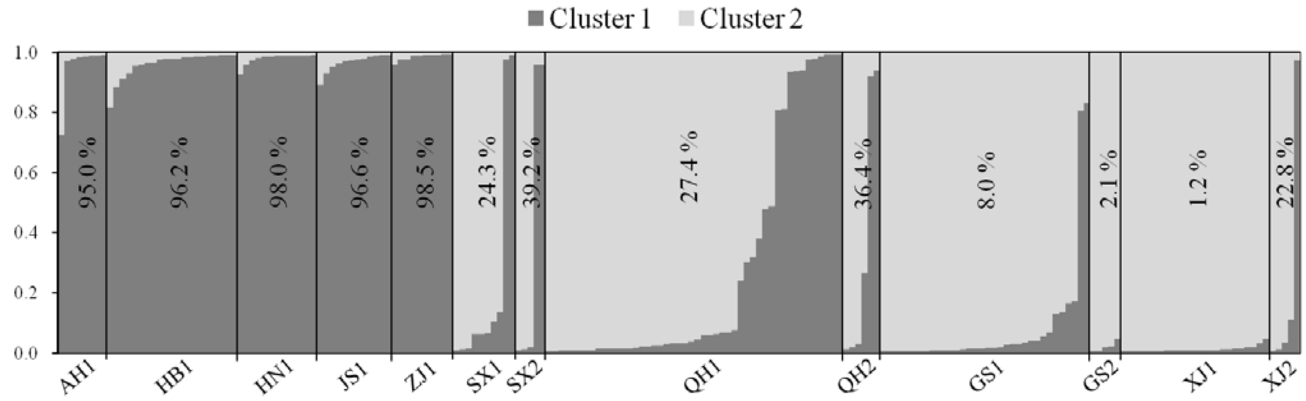

Supplement: Supplementary file 1 [file insects-11-00117-s001.pdf]
